# Effect of non-surgical periodontal treatment on transferrin serum levels in patients with chronic periodontitis

**DOI:** 10.15171/joddd.2016.027

**Published:** 2016-08-17

**Authors:** Adileh Shirmohamadi, Mohamad Taghi Chitsazi, Masoumeh Faramarzi, Ashkan Salari, Fereshteh Naser Alavi, Nazila Pashazadeh

**Affiliations:** ^1^Professor, Department of Periodontics, Faculty of Dentistry, Tabriz University of Medical Sciences, Tabriz, Iran; ^2^Associate Professor, Department of Periodontics, Faculty of Dentistry, Tabriz University of Medical Sciences, Tabriz, Iran; ^3^Postgraduate Student, Department of Periodontics, Faculty of Dentistry, Tabriz University of Medical Sciences, Tabriz, Iran; ^4^Postgraduate Student, Department of Operative Dentistry, Faculty of Dentistry, Tabriz University of Medical Sciences, Tabriz, Iran; ^5^Nurse, Department of Periodontics, Faculty of Dentistry, Tabriz University of Medical Sciences, Tabriz, Iran

**Keywords:** Transferrin, chronic periodontitis, inflammation, dental scaling, root planing

## Abstract

***Background.*** Transferrin is a negative acute phase protein, which decreases during inflammation and infection. The aim of the present investigation was to evaluate changes in the transferrin serum levels subsequent to non-surgical treatment of chronic periodontal disease.

***Methods.*** Twenty patients with chronic periodontitis and 20 systemically healthy subjects without periodontal disease, who had referred to Tabriz Faculty of Dentistry, were selected. Transferrin serum levels and clinical periodontal parameters (pocket depth, clinical attachment level, gingival index, bleeding index and plaque index) were measured at baseline and 3 months after non-surgical periodontal treatment. Data were analyzed with descriptive statistical methods (means ± standard deviations). Independent samples t-test was used to compare transferrin serum levels and clinical variables between the test and control groups. Paired samples t-test was used in the test group for comparisons before and after treatment. Statistical significance was set at P < 0.05.

***Results.*** The mean transferrin serum level in patients with chronic periodontitis (213.1 ± 9.2 mg/dL) was significantly less than that in periodontally healthy subjects (307.8 ± 11.7 mg/dL). Three months after periodontal treatment, the transferrin serum level increased significantly (298.3 ± 7.6 mg/dL) and approached the levels in periodontally healthy subjects (P < 0.05).

***Conclusion.*** The decrease and increase in transferrin serum levels with periodontal disease and periodontal treatment, respectively, indicated an inverse relationship between transferrin serum levels and chronic periodontitis.

## Introduction

Periodontitis is a chronic inflammatory condition with a polymicrobial infectious nature, which destroys the tooth-supporting tissues and results in attachment and bone loss. After accumulation of bacterial biofilm on the external tooth surfaces, periodontitis is induced as an inflammatory‒immune process and results in the production of proinflammatory cytokines, local and systemic inflammatory responses, and formation of periodontal pockets.^1^ The diagnosis of periodontitis relies on clinical measurements, including pocket depth, clinical attachment level, and radiographic findings. However, such measurements have limited efficacy in periodontal diagnosis in many cases because these parameters mainly indicate previous periodontal disease rather than the current disease activity.^2^

It is necessary to develop new diagnostic tests so that it would be possible to diagnose active disease, predict future disease activity and evaluate response to periodontal treatment after clinical recovery of patients from periodontitis. Advances in relation to the diagnosis of periodontal diseases include moving toward diagnostic techniques that can evaluate periodontal disease risk by measuring biomarkers. Biomarkers are produced by both healthy individuals and those with specific systemic conditions. They are molecules that can be used to monitor the health status, disease initiation and response to treatment.^3^

Acute phase proteins are some of these biomarkers. The acute phase reaction consists of a group of metabolic and systemic changes that are induced by an inflammatory stimulus. The most critical components of this reaction are acute phase proteins that consist of a heterogeneous group of serum proteins synthesized by the liver.^4^

A change in the concentration of acute phase proteins, as a patho-physiologic phenomenon, occurs subsequent to a wide range of disorders, including inflammation, trauma, infection and infarction. Despite their name, such proteins are expressed due to both acute and chronic inflammation.^5^

Acute phase proteins are categorized into two groups:

1. Positive acute phase proteins such as C-reactive protein, serum amyloid-A, pentraxin-3, ceruloplasmin and ferritin, whose serum levels increase during the inflammatory response.

2. Negative acute phase proteins are proteins such as albumin and transferrin, whose serum levels decrease with an increase in inflammation severity.^6^

Transferrin is an acute phase protein, whose serum levels decrease during inflammation. It is a glycoprotein which is involved in the following functions: transfer of iron in the blood to all the body parts, a role in innate immunity and a bactericidal effect on bacteria. The serum transferrin is measured for various reasons: to determine the cause of anemia, to evaluate iron metabolism (for example, in iron deficiency anemia) and to determine the iron-carrying capacity of the blood. Low serum transferrin can disturb hemoglobin production, leading to anemia. Both poor production of transferrin by the liver (where it is synthesized) and excessive loss of transferrin through the kidneys into the urine can lead to low transferrin serum levels. A number of conditions such as infection and malignancy can decrease transferrin levels.^7^

There is a clear relationship between inflammation, immune cell mediators, release of acute phase proteins and bacteria-induced cytokines and periodontal tissues. Periodontal diseases result in an increase in systemic inflammatory responses and changes in the serum levels of biomarkers.^8^Studies have confirmed changes in the serum levels of acute phase proteins in patients with chronic periodontitis. Slade et al^9^ and Megson et al^10^ reported increased serum levels of C-reactive protein (CRP) in patients with periodontitis. Chakraborty et al^11^ showed an increase in plasma ferritin levels in the chronic periodontitis group. A study by Harshavardhana et al^12^ showed an increase in serum levels of ceruloplasmin in both chronic and aggressive periodontitis, with greater changes in patients with aggressive periodontitis. Gümüş et al^13^ reported an increase in serum levels of pentroxin-3 in both generalized chronic and aggressive periodontitis. Iwasaki et al^14^ reported an inverse relationship between periodontitis and albumin serum levels. In addition, studies have shown that treatment of severe periodontitis can result in changes in the serum levels of acute phase proteins. Based on the results of studies by Radafshar et al,^15^ George et al,^16^ and Anne et al,^17^ non-surgical periodontal treatment in patients with severe periodontitis resulted in a decrease in serum levels of CRP. A study by Chakaborty et al^11^ showed a decrease in ferritin serum levels 3 months after non-surgical periodontal treatment. However, no study is available on the relationship between transferrin serum levels and chronic periodontitis and non-surgical periodontal treatment in systematically healthy subjects.

Based on the observations above, we hypothesized that a decrease in transferrin serum levels might be related to chronic periodontitis and a return to its normal levels can be expected in response to periodontal treatment. Therefore, the present study was undertaken to evaluate changes in the serum levels of transferrin in patients with periodontal disease and periodontally healthy subjects, before and after non-surgical treatment, and its relationship with clinical periodontal parameters.

## Methods

The study protocol was approved by the Ethics Committee of Tabriz University of Medical Sciences under the code TBZMED.REC.1394.870.

The subjects in the present study were selected from the patients referring to the Department of Periodontics, Tabriz Faculty of Dentistry, Iran. In a pilot study, carried out before the present study, the serum levels of transferrin were measured in 5 healthy subjects as controls and in 5 subjects with chronic periodontitis at baseline and 3 months after non-surgical periodontal treatment and based on the results at least 16 subjects were included in each group at α = 0.05 and a statistical power of 80%. The study population consisted of 40 subjects with an age range of 25‒56 years (20 males and 20 females). All the subjects were systemically healthy, had no smoking habits and had at least 20 permanent teeth in their oral cavity.

The study included two groups: 20 in the control group, healthy from a periodontal point of view, with no attachment loss and no pockets with a probing depth of ≥3 in any area in any tooth; the bleeding index in the whole oral cavity was ≤10%;^18^ the test group consisted of 20 patients with chronic periodontitis, selected based on inclusion criteria: at least 2 interproximal areas with an attachment loss of ≥4 mm or with at least 2 interproximal areas with a pocket depth of ≥5 mm but not in the same tooth.^19^

A definitive diagnosis of chronic periodontitis was established based on American Academy of Periodontology (AAP) classification with the use of the clinical parameters of pocket depth, attachment loss and radiographic evidence of bone loss.^20^

The exclusion criteria consisted of a history of taking nonsteroidal anti-inflammatory drugs (NSAIDs) and any antimicrobial agent during a 3-month period before the study, a history of any periodontal treatment (surgical and non-surgical) during a 6-month period before the study, use of mouthrinses and vitamin supplements during a 3-month period before the study, pregnancy or breastfeeding, any systemic disease, any infectious disease other than chronic periodontitis, and presence of aggressive periodontitis. All the risks and advantages of this clinical study were explained to the subjects and informed consent forms were obtained from all the subjects.

Venous blood samples (4 mL) were collected from all the subjects at baseline (before recording the clinical parameters) by professional operators. The blood samples were transferred into sterile vacuum tubes with no anticoagulant and sent to the laboratory in less than 2 hours. An automated analyzer (Minday Co., Model BS-480, China) was used to determine serum levels of transferrin using an enzymatic technique.

The periodontal status of the whole oral cavity in all the subjects was evaluated and recorded by determining the pocket depths, attachment loss at 6 areas in each tooth except for third molars (mesiolingual, lingual, distolingual, distobuccal, buccal and mesiobuccal), bleeding index (Lenox & Kopczyk),^21^ gingival index and plaque index (Sillness & Löe)^22,23^ by one operator. Therefore, there was no variation in the operator. To determine the reproducibility of the examination, each clinical parameter was recorded twice in one session at a 1-hour interval in 10 subjects. Evaluation of mean differences between the values showed an accuracy rate of 90% with a Kappa value of 0.81.

Non-surgical periodontal treatment in the chronic periodontitis group consisted of oral hygiene instruction and meticulous SRP and administration of 0.12 % chlorhexidine (CHX) mouthwash twice daily for 30 seconds for 2 weeks (Kin Gingival Mouthwash, Cosmodent Co., Spain). SRP was administrated by one operator in a 1-hour session. The subjects in the control group only received oral hygiene instructions. The subjects in the test group were followed at 4-, 8- and 12-week intervals after periodontal treatment for the evaluation of the maintenance status of oral hygiene. After 3 months of follow-up, venous blood samples were once again collected from the subjects and then the clinical parameters were re-evaluated.

All the statistical analyses were carried out with SPSS 20. Statistical significance was set at P < 0.05. Kolmogorov-Simonov test was used to evaluate normal distribution of data, which showed that all the data were distributed normally; therefore, parametric tests were used to evaluate mean differences between the groups. Data were analyzed with descriptive statistics means ± standard deviations).

Independent-samples t-test was used to compare transferrin serum levels and clinical parameters between the two groups. Paired-samples t-test was used in the test group for comparisons before and after treatment. Spearman’s rank correlation coefficient was used to determine the statistical significance of correlation between clinical and biochemical parameters.

## Results

A total of 40 subjects were selected (20 males and 20 females) from those referring to the Department of Periodontics based on inclusion and exclusion criteria in two groups. Twenty periodontally healthy subjects (9 males and 11 females) with an age range of 25‒42 years and a mean age of 33.1 ± 4.14 years were assigned to the control group. The test group consisted of twenty patients with chronic periodontitis (11 males and 9 females) with an age range of 28‒56 years and a mean age of 42.13 ± 8.36 years. The subjects in the test group had generalized moderate-to-severe chronic periodontitis. None of the participants were excluded from the study during the study period.

Table 1 presents the descriptive clinical and biochemical parameters of the subjects at baseline. There were significant differences in PD, CAL, GBI, GI and P between the test and control groups (P < 0.05). The mean serum transferrin levels in the control and chronic periodontitis groups were 307.8 ± 11.7 and 213.1 ± 9.2 mg/dL, respectively, with significant differences between the two groups.

**Table 1:** Descriptive clinical and biochemical parameters of the subjects at baseline

**Parameters**	**Total**	**Chronic periodontitis**	**Periodontally healthy**	**P-value**
**PD (mm)**	3.47 ± 1.62	5.03 ± 0.48	1.92 ± 0.33	<0.001^*^
**CAL (mm)**	2.73 ± 2.45	5.13 ± 0.47	0.55 ± 0.12	<0.001‏^*^
**GBI**	39.49 ± 36.02	74.66 ± 4.33	7.86 ± 1.92	<0.001‏^*^
**GI**	1.33 ± 0.87	2.17 ± 0.24	0.50 ± 0.13	<0.001^*^
**PI**	1.28 ± 0.90	2.13 ± 0.31	0.79 ± 0.13	<0.001‏^*^
**Serum transferrin )mg/dL)**	260.90 ± 49.57	213.1 ± 9.2	307.8 ± 11.7	<0.001^*^

‏^*^Statistically significant (P < 0.05) PD: probing depth; CAL: clinical attachment level; GBI: gingival bleeding index; GI: gingival index; PI: plaque index

Table 2 presents the Spearman’s rank correlation coefficients. There was no significant correlation between the serum levels of transferrin and clinical periodontal parameters.

**Table 2:** Spearman’s rank correlation coefficients (statistical significance) for the studied parameters

**Parameter**	**PD**	**CAL**	**GBI**	**GI**	**PI**	**Serum** **transferrin**
**PD**	—	0.99 (0.000)^*^	0.518 (0.019)^*^	0.470 (0.036)^*^	0.642 (0.002)†	−0.305 (0.190)
**CAL**	—	—	0.493 (0.027)^*^	0.427 (0.060)	0.612 (0.004)†	−0.323 (0.165)
**GBI**	—	—	—	0.465 (0.039)^*^	0.344 (0.138)	−0.256 (0.276)
**GI**	—	—	—	—	0.457 (0.043)^*^	−0.457 (0.043)
**PI**	—	—	—	—	—	−0.040 (0.866)
**Serum transferrin**	—	—	—	—	—	—

^*^Correlation is significant at the 0.05 level (2-tailed) †Correlation is significant at the 0.01 level (2-tailed) PD: probing depth; CAL: clinical attachment level; GBI: gingival bleeding index; GI: gingival index; PI: plaque index

Three months after non-surgical periodontal treatment, the PD, CAL, GBI, PI and GI values improved (Table 3). The mean differences between all the clinical parameters were significant between the baseline and after non-surgical periodontal treatment (P < 0.001).

**Table 3:** Clinical periodontal parameters 3 months after non-surgical periodontal treatment

**Parameters**	**Before periodontitis treatment**	**After periodontitis treatment**	**P-value**
**PD (mm)**	5.03 ± 0.48	3.29 ± 0.27	<0.001^*^
**CAL (mm)**	5.13 ± 0.47	3.87 ± 0.91	<0.001^*^
**GBI (%)**	74.66 ± 4.33	17.13 ± 4.07	<0.001^*^
**GI**	2.17 ± 0.24	0.98 ± 0.31	<0.001^*^
**PI**	2.13 ± 0.31	0.81 ± 0.30	<0.001^*^
**Serum transferrin (mg/dL)**	213.1 ± 9.2	298.3 ± 7.6	<0.001^*^

^*^Statistically significant (P < 0.05).

All the subjects with chronic periodontitis exhibited significant increases in transferrin serum levels 3 months after periodontal treatment (P < 0.01). In addition, the transferrin serum levels after intervention (298.3 ± 7.6 mg/dL) approached the levels in the control group (307.8 ± 11.7 mg/L).

## Discussion

In the present study, the effects of periodontal disease and non-surgical periodontal treatment on transferrin serum levels were evaluated. Based on the results, the transferrin serum levels in patients with chronic periodontitis were significantly lower than those in periodontally healthy subjects. In addition, a decrease in periodontal inflammation after non-surgical periodontal treatment resulted in an increase in transferrin serum levels up to those in healthy controls.

Chronic periodontitis leads to the loss of connective tissue attachment and bone supporting the teeth through accumulation of subgingival anaerobic gram-negative bacteria.^24^ Changes take place in the serum levels of acute phase proteins in response to bacterial infection, which result in the synthesis of IL-1, IL-6 and TNF-α. These proinflammatory cytokines have a main role in the destruction of periodontal tissues. The response mounted by acute phase proteins affects the defensive and adaptive mechanisms that occur before the immunologic response.^25,26^ These proteins are synthesized by hepatocytes and are categorized into the positive and negative acute phase proteins based on changes in their concentrations. One of these negative acute phase proteins is transferrin, which is a blood plasma protein that binds firmly but reversibly to iron and carries it in the plasma. In addition, it has a role in the innate immunity. Transferrin binds to Fe^3+^ and creates an environment with a low concentration of iron, in which only a small number of bacteria can survive. Therefore, transferrin has a bactericidal effect.^7^

Some studies are available on the relationship between positive acute phase proteins and periodontal diseases, all of which have confirmed a direct relationship between the positive acute phase proteins and periodontitis.^9-13^ However, a very small number of studies are available on the relationship between negative acute phase proteins and periodontal disease. Iwasaki et al^14^evaluated albumin, a negative acute phase protein, in patients with periodontitis and concluded that there might be an inverse relationship between periodontitis and albumin serum levels. In the present study, there was an inverse relationship between transferrin serum levels and chronic periodontics, which might be consistent with the results reported by Iwasaki et al, indicating an inverse relationship between periodontitis and negative acute phase proteins.

Only a few studies are available on the relationship between transferrin serum levels and periodontal disease. Rodrigues et al^27^evaluated the serum levels of biomarkers in patients undergoing hemodialysis in two groups of periodontally healthy and chronic periodontitis subjects. The transferrin serum levels were lower in the subjects with chronic periodontitis but the differences between the two groups were not significant. Since the study above did not include systemically healthy subjects, its results cannot be compared with those of the present study.

One reason for a decrease in serum transferrin in patients with chronic periodontitis compared to the controls might be the effects of chronic inflammation on systemic conditions. It appears TNF-α, IL-1 and IL-6 are critical down-regulators for the production of this acute phase protein.^28,29^

Iron is necessary for the growth and metabolism of almost all the microorganisms. Transferrin is a glycoprotein with two sites for binding iron and it might be a significant source of iron for pathogens.^30^

Of all the periodontopathogens, *Porphyromonas gingivalis* is a gram-negative anaerobic bacterial species, which has a role as a main etiologic agent in the initiation and progression of chronic periodontitis. *P. gingivalis* can destroy tissues and plasma proteins through the synthesis of endopeptidases referred to as gingipains.^31^ Another reason for a decrease in transferrin serum levels in chronic periodontitis might be the ability of *P. gingivalis* to cleave transferrin and acquire iron from transferrin during disease. *P. gingivalis* results in the proteolytic destruction of transferrin through gingipain which is a cysteine protease and an important virulence factor for it; therefore, *P. gingivalis* can acquire iron which is necessary for its long-term growth.^32^ In addition, *Campylobacter rectus* and *Prevotella intermedia* use transferrin as a source of iron for their growth.^33-34^ Therefore, the mechanism above can explain the decrease in transferrin serum levels subsequent to chronic periodontitis and an increase in the counts of periodontopathogens.

A few studies are available on the effect of non-surgical periodontal treatment on positive acute phase proteins. Anne et al^17^ and Radafshar et al^15^ evaluated the effect of non-surgical treatment of periodontitis on the serum levels of these proteins in severe periodontitis group and reported that periodontal intervention diminished the serum levels of CRP. Grazianiet al^35^ reported a return of fibrinogen serum levels to normal levels after non-surgical periodontal treatment in patients with generalized chronic periodontitis. Chakraborty et al^11^ showed that non-surgical periodontal treatment in patients with generalized chronic periodontitis resulted in a decrease in ferritin serum levels. However, no study is available on the effect of non-surgical periodontal treatment on the negative acute phase proteins.

The present study is the first interventional study to report the effect of periodontal treatment on transferrin serum levels. Three months after periodontal treatment, improvements were detected in all the periodontal clinical parameters in association with an increase in transferrin serum levels in patients with chronic periodontitis. This finding might be attributed to a decrease in proinflammatory cytokine levels, especially IL-6, after periodontal intervention in patient with chronic periodontitis since IL-6 is the chief agent for the hepatocytic secretion of the majority of acute phase proteins.^7^

It appears logical that if periodontal inflammation decreases transferring serum levels, these levels should increase subsequent to non-surgical periodontal treatment which decreases the load of systemic inflammation.

The present study was a preliminary study and it is necessary to evaluate the correlation between transferrin serum levels and the serum levels of other inflammatory cytokines so that the relationship between inflammation and transferrin serum levels can be further elucidated.

Further studies are necessary with larger sample sizes to determine whether transferrin serum levels can be used as diagnostic markers for periodontal diseases.

## Conclusion

The results of this study showed an inverse relationship between serum transferrin and chronic periodontitis, i.e. lower transferrin serum levels were detected in patients with chronic periodontitis. Non-surgical periodontal treatment resulted in an increase in serum levels of transferrin in chronic periodontitis group, which reached the levels in periodontally healthy subjects.

## Acknowledgments

The authors would like to acknowledge the Dental and Periodontal Research Center at Tabriz University of Medical Sciences for the financial support of this project.

## Authors’ contributions

The study was planned by AS and AS. AS carried out the study experiments. NP and AS collected blood samples from the patients. The statistical analyses and interpretation of data were carried out by MF. AS, MTC, FNA and AS contributed to the literature review. All the authors have read and approved the final manuscript.

## Funding

The study was supported by Dental and Periodontal Research Center at Faculty of Dentistry, Tabriz University of Medical Sciences.

## Competing interests

The authors declare that they have no competing interests with regards to authorship and/or publications of this paper.

## Ethics approval

The study protocol was approved by the Ethics Committee of Tabriz University of Medical Sciences under the code TBZMED.REC.1394.870.
