# Erratum to: Effect of non-surgical periodontal treatment on transferrin serum levels in patients with chronic periodontitis

**DOI:** 10.15171/joddd.2016.044

**Published:** 2016-12-21

**Authors:** Adileh Shirmohammadi, Mohamad Taghi Chitsazi, Masoumeh Faramarzi, Ashkan Salari, Fereshteh Naser Alavi, Nazila Pashazadeh

**Affiliations:** ^1^Professor, Department of Periodontics, Faculty of Dentistry, Tabriz University of Medical Sciences, Tabriz, Iran; ^2^Associate Professor, Department of Periodontics, Faculty of Dentistry, Tabriz University of Medical Sciences, Tabriz, Iran; ^3^Postgraduate Student, Department of Periodontics, Faculty of Dentistry, Tabriz University of Medical Sciences, Tabriz, Iran; ^4^Postgraduate Student, Department of Operative Dentistry, Faculty of Dentistry, Tabriz University of Medical Sciences, Tabriz, Iran; ^5^Nurse, Department of Periodontics, Faculty of Dentistry, Tabriz University of Medical Sciences, Tabriz, Iran

 In the article entitled “Effect of non-surgical periodontal treatment on transferrin serum levels in patients with chronic periodontitis” which appeared in* J Dent Res Dent Clin Dent Prospect* 2016; 10(3):169-175, the first author’s name was misspelled. The correct name of the first author is Adileh Shirmohammadi. The journal regrets this error.

